# Exploration of pathological prediction of chronic kidney diseases by a novel theory of bi-directional probability

**DOI:** 10.1038/srep32151

**Published:** 2016-08-25

**Authors:** Yuan Yang, Min Luo, Li Xiao, Xue-jing Zhu, Chang Wang, Xiao Fu, Shu-guang Yuan, Fang Xiao, Hong Liu, Zheng Dong, Fu-you Liu, Lin Sun

**Affiliations:** 1 Department of Nephrology, The Second Xiangya Hospital, Central South University, Changsha, Hunan 410011, China; 2 Dong Medicine Key laboratory of Hunan Province, Department of laboratory medicine, Hunan University of Medicine, Huaihua, Hunan 418000, China; 3 Department of Health Toxicology, Xiangya School of Public Health, Central South University, Changsha, Hunan 410008, China

## Abstract

In the clinic, the pathological types of chronic kidney diseases (CKD) are considered references for choosing treatment protocols. From a statistical viewpoint, a non-invasive method to predict pathological types of CKD is a focus of our work. In the current study, following a frequency analysis of the clinical indices of 588 CKD patients in the department of nephrology, a third-grade class-A hospital, a novel theory is proposed: “bi-directional cumulative probability dichotomy”. Further, two models for the prediction and differential diagnosis of CKD pathological type are established. The former indicates an occurrence probability of the pathological types, and the latter indicates an occurrence of CKD pathological type according to logistic binary regression. To verify the models, data were collected from 135 patients, and the results showed that the highest accuracy rate on membranous nephropathy (MN-100%), followed by IgA nephropathy (IgAN-83.33%) and mild lesion type (MLN-73.53%), whereas lower prediction accuracy was observed for mesangial proliferative glomerulonephritis (0%) and focal segmental sclerosis type (21.74%). The models of bi-directional probability prediction and differential diagnosis indicate a good prediction value in MN, IgAN and MLN and may be considered alternative methods for the pathological discrimination of CKD patients who are unable to undergo renal biopsy.

Chronic kidney disease (CKD) is a common chronic disease that occurs in the elderly population and presents with the characteristics of an estimated glomerular filtration rate of <60 mL/min per 1.73 m^2^ or a urinary albumin: creatinine ratio of >30 mg/g^1^. Generally, CKD is classified as one of two main types in the clinical setting: chronic nephritis or nephrotic syndrome. Indications include oedema, high blood pressure, proteinuria, haematuria, hypoalbuminemia, hyperlipidaemia, and changes in urine. In addition, CKD can be present in a complex form, such as lupus nephritis, purpura nephritis, hypertensive nephropathy, diabetic nephropathy, obstructive nephropathy, vasculitis related kidney disease, and allergic interstitial nephritis due to the diversity of primary diseases or inducements. The discrimination of CKD pathological types is important for treatment in the clinic. The common types are described as a mild lesion type (MLN), focal segmental sclerosis type (FSGS), membranous nephropathy (MN), IgA nephropathy (IgAN), and mesangial proliferative glomerulonephritis (MsPGN).

However, the present method of identifying CKD pathological type is through a renal y biopsy, which necessitates certain surgical procedures and specific histochemical staining. Under some conditions, it is not feasible to perform a renal biopsy in CKD patients due to clinical condition or non-adherence. Statistics can be used to evaluate the regularity of CKD pathological types associated with clinical data in CKD patients, such as gender, age, occupation, disease duration, inducements, first medication, clinical signs, and the laboratory results of blood or urine assays. Currently, the statistical study of predicting pathological types of CKD patients is still a new field, which is helpful for clinical diagnosis and treatment. In the current study, to determine whether the CKD pathological type is associated with the frequency of clinical signs of CKD patients, the clinical data of CKD patients, including their basic characteristics, medical history, results of clinical and laboratory exams, were analyzed. A novel theory, the “bi-directional cumulative probability dichotomy” is proposed, and two models for the prediction and differential diagnosing of CKD pathological type are established. Further, we apply the CKD sample to verify the accuracy rate of models.

## Results

### Frequency of clinical indexes in CKD patients with a definite pathology diagnosis

According to the database of CKD pathological classification, the frequency of each clinical index in different CKD pathological types is detected by calculating the ratio of index’ count vs total count, respectively. For example, the frequency of “Proteinuria <2Y” in MN is highly as 87.9%, while that is relative low as 70.1%. These subtle differences between them indicated the different characteristic of each clinical index in the corresponding CKD pathological types. The frequency of clinical indices in CKD pathological types was shown in the Table 1.

### Theory of bi-directional probability accumulation dichotomy

#### Principles of bi-directional probability accumulation

##### Theory of bi-directional probability accumulation

**Figure 1:**
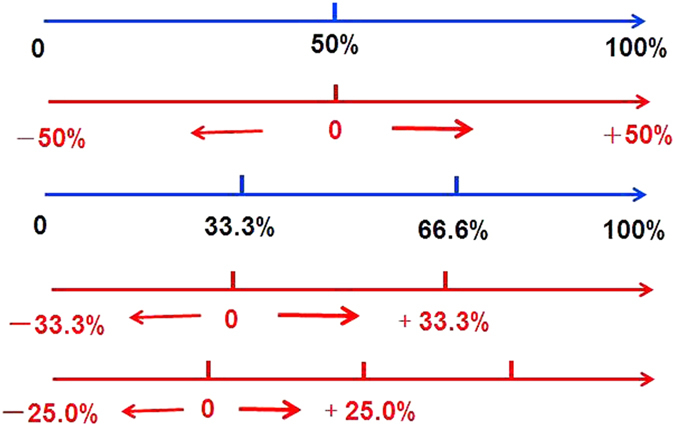
Theory of bi-directional probability.

According to the principle of detecting frequency and the probability theory, the different frequencies of clinical indices in the χ^2^ test were distributed in different CKD pathological types. If the frequency was as high as *P* > 0.50 (binominal distribution), *P* > 0.33 (trinomial distribution), or *P* > 0.25 (quadrinomial or higher distribution), the frequencies were defined as having a positive probability (p1), which was demonstrated as *p1*-0.5/*p1*-0.33/*p1*-0.25. In contrast, if the frequency was as low as *P* < 0.50 (binominal distribution), *P* < 0.33 (trinomial distribution), or *P* < 0.25 (quadrinomial or higher distribution), the frequencies were defined as having negative probability (p2), which was demonstrated as 0.5-*p2*/0.33-*p2*/0.25-*p2*. Further, the positive and negative probabilities were summed as ∑positive (P1) or ∑negative (P2), respectively, and the difference between the positive probability and the negative probability was defined as the excess probability of one CKD pathological type, and the corresponding formula is ∑positive (P1)-∑negative (P2). For visualizing the concept, an imagistic perception is shown in Fig. 1.

##### Principle of dichotomy

The above concept of bi-directional probability accumulation was based on the assumption of a certain CKD pathological type. Generally, we assume that the occurrence of any one CKD pathological type is a random event, so the basic probability of any one CKD pathological type is 50%. Therefore, the predictive probability of CKD pathological type is deduced as ∑positive (P1) -∑negative (P2)+50%.

#### Estimation of sampling error of ∑positive (P1) or ∑negative (P2)

Because p1/p2 is calculated from the sample data of one pathological type, the sampling error of p1 or p2 should be estimated in the model of bi-directional probability accumulation. For the accumulated value of bi-directional probability ∑positive (P1) or ∑negative (P2), the sampling error was calculated as ΣP1 ± [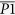 × (1 − 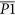)/n]^1/2^ ; ΣP2 ± [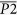 × (1 − 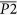)/n]^1/2^

#### Establishment of the bi-directional probability accumulation dichotomy formula

Following the detection and analysis of bi-directional frequency in different pathological types, the principle of bi-directional probability accumulation dichotomy is applied to establish the prediction model of CKD pathological types, and the predictive formula was expressed as follows:

ΣP1 ± [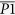 × (1 − 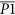)/n]^1/2^ − ΣP2 ± [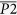 × (1 − 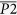)/n]^1/2^+50%. Based on the predictive formula of CKD pathological types, the occurrence probability of CKD pathological types can be calculated by analyzing “yes” or “no” responses to the clinical indexes in one CKD patient. The indices of positive or negative probability (p1 or p2) in 5 pathological types (see Supplement information)

#### Logistic regression model for differential diagnosis of CKD pathological types

Following the establishment of the bi-directional probability accumulation dichotomy, the predictive probability of CKD pathological types can be calculated by the responses of “yes” or “no” to clinical indexes in CKD patients. The results of two adjacent probabilities in a patient will be deduced, so the solution is the establishment of a logistic regression model of differential diagnosis. Six pairs of logistic regression models of differential diagnosis were established between each two CKD pathological types (MLN vs FSGS; IgAN vs FSGS; IgAN vs MLN; IgAN vs MN; MN vs MLN; FSGS vs MN). Based on the OR (odds ratio) in the 6 pairs of models of CKD pathological types, we set up a discrimination method of any one CKD pathological type. First, the propensity factors of each CKD pathological type are calculated and summed as ∑OR1 (prediction group) or ∑OR2 (control group); if the difference value of ∑OR1 - ∑OR2 is > 0, then the judgement is the OR1-indicated pathological type. Conversely, if the difference value of ∑OR1 - ∑OR2 is < 0, then the judgement is the OR2-indicated pathological type. In addition, due to a small sample number and the fact that models of MsPGN were not established, details are shown in Supplement information.

#### Verification of models of probability prediction and differential diagnosis

To verify the accuracy of the probability prediction and differential diagnosis models, the verifying software in the Excel program was applied to examine the CKD pathological types. Predicting the results of 135 CKD patients showed that the highest accuracy rate was obtained using MN (100%), followed by IgAN (83.33%) and MLN (73.53%), and a lower prediction accuracy was found for MsPGN (0%) and FSGS (21.74%). However, the MN prediction also demonstrated a relatively high rate of false judgment, as detailed in Table 2.

## Discussion

CKD is a common chronic disease in the general population, and its prevalence is estimated to range from 10.2% to 13.0% in the United States, Norway and other countries^2^,^3^. An epidemiological survey showed that the prevalence of CKD is as high as 10.8% in China^4^; the number of CKD cases in China is approximately 147.96 million according to the estimation of the overall population of China of 1.37 billion (data from the sixth national population census in 2010), especially for the primary kidney diseases such as primary glomerulonephritis, primary nephrotic syndrome, that are frequent occurred and showed the typical clinical features of proteinuria, hematuria, edema, high blood pressure, etc. At present, the first treatment of primary kidney diseases is very important to subsequent development of nephritic pathology, which is also an important factor that affects the outcome of end-stage renal disease (ESRD). Consequently, patients with ESRD are required to undergo expensive renal replacement therapy (e.g., haemodialysis or peritoneal dialysis or renal transplantation), resulting in a great reduction the quality of life of these patients.

Currently, the accurate judgment of pathological type is mainly dependent on renal biopsy. Clinically, not all patients are willing to undergo a traumatic operation for renal biopsy, or they may exhibit clinical contraindications for renal biopsy. In cases the above situation occurs, the pathological discrimination of CKD patients is difficult, which can lead to confusion in choosing reasonable treatments for patients. Therefore, the present of study, we focused on the accurate prediction of the various pathological types in CKD patients with primary glomerular disease by statistical theory, and expect to be applied in the clinic, and to achieve the goal of effectively controlling CKD and delaying the onset of ESRD by the appropriate treatment of patients as much as possible.

In the current study, following the frequency detection of clinical indices of CKD patients with primary glomerular disease and the characteristics of different pathological types are analyzed, the theory of “bi-directional probability accumulation dichotomy” is proposed, and the judgment of relative pathological types is achieved by calculating the total value of bi-directional probability. Specifically, the probability value of predicting the pathological types of the primary glomerular disease can be calculated by collecting the frequency of “yes” answers to matching clinical indexes, such as basic characteristics, medical history, signs and laboratory results for a CKD patient with primary kidney disease. Therefore, based on the theory of “bi-directional probability accumulation dichotomy”, the calculating formula was expressed as follows: ΣP1 ± [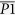 × (1 − 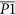)/n]^1/2^ − ΣP2 ± [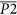 × (1 − 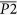)/n]^1/2^+50%. Further, when the predictive probabilities of the relative pathological types were similar, the 6 pair models of differential diagnosis were established, and the judgement method of CKD pathological type was established, detailed as logistic regression models of MLN vs FSGS, FSGS vs IgAN, IgAN vs MLN, IgAN vs MN, MN vs MLN, and MN vs FSGS.

To apply them in the clinic, the theory of “bi-directional probability accumulation dichotomy” and the models of differential diagnosis must be validated in practice. Software for probability prediction and differential diagnosis was designed and applied to verify the prediction accuracy of the above models. The results showed the highest accuracy rate for MN (100%), followed by IgAN (83.33%) and MLN (73.53%), whereas lower prediction accuracy was shown for MsPGN (0%) and FSGS (21.74%). The results indicated a good prediction value for MN, IgAN and MLN patients. However, the prediction value of MN also showed a relative high rate of false judgment in non-MN patients. In conclusion, the present of study provides a preliminary reference for predicting the pathological types of CKD patients with primary glomerular diseases by a non-invasive method.

Although we got some useful results from this study, the limitations of sample number and prediction accuracy in this study should be considered in future studies. Especially, increasing the prediction accuracy on FSGS or MsPGN and decreasing the misjudgement rate on MN will be a key focus. Therefore, some substantial improvements need to be carried out in next study, such as extending the scope of collecting CKD patients from more hospitals to increase sample number, In addition, some rare pathological types in adult of China, such as sclerosing glomerulonephritis, minimal change nephrosis and MsPGN, which expect to be included in the next improved model.

Indeed, the establishment of a comprehensive prediction system is a very valuable for the application in clinic. Noticeably, the present model aim only to the prediction of the pathological types in primary glomerular diseases. For the secondary kidney diseases such as lupus nephritis, amyloidosis, diabetic nephropathy, which have complex clinical characteristic and are identified by clinical various examinations. Thus, another corresponding model should be also established based on other statistical viewpoint in future study. Of course, the later model and the former model should be closely associated and cooperated, together for applying in predicting various kidney diseases pathologic types in clinical.

Summary, the current research is only an exploration and an attempt to predict the pathological types of primary glomerular diseases from a statistical probability perspective. Our ultimate goal is to establish a complete system of probability prediction on the pathological types in various kidney diseases which is applied in the prediction of patients with kidney diseases and uncovers a new field of prediction medicine for internal medicine.

## Materials and Methods

### Collection of clinical data in patients with CKD

Data were obtained from the archives of 723 CKD cases, including 588 cases for the establishment of models and 135 cases for the verification of models. Archives were from patients seen from July 2011 to August 2014 at the Department of Nephrology at a third-grade class-A hospital. The study was classified as statistics and was approved by medical ethics committee of the Second Xiangya Hospital in China. The criteria for case selection included the following: **A.** A histopathologic diagnosis according to biopsy and clinical diagnosis is clear; **B.** Patient age is from 13 to 65 years old, the length of hospital stay was ≥4 days, and the detailed medical history records and clinical test results were available; **C**. Clinical CKD staging referred to 2012 KDIGO guidelines (chronic kidney disease assessment and management). Patients in the G1 stage account for the predominant proportion of CKD patients, and all patients were in stages ≤ G4 (corrected by the China MDRD formula: c-aGFR(ml/min/1.73 m^2^) = 186 × [Cr]^−0.203^ × [age]^−1.154^ × [female × 0.742] × 1.233)^5^,^6^.

The criteria for case exclusion included the presence of end-stage renal disease or long-term renal replacement therapy such as haemodialysis or peritoneal dialysis. A total of 588 cases were used to build prediction models, and 120 patients were classified as having mild lesion nephrosis (MLN); 122 patients were diagnosed with focal segmental glomerularsclerosis (FSGS); 141 with membranous nephropathy (MN); 137 with IgA nephropathy-IgAN (these IgAN cases were further divided into IgA-mild lesion (IgA-ML) and IgA-focal segmental sclerosis or focal sclerosis or Hyperplasia with sclerosis (IgA-FS)); 9 patients with sclerosing glomerulonephritis (SGN); 27 and with mesangial proliferative glomerulonephritis (MsPGN). After the models were constructed, the remaining 135 CKD cases were used to verify the accuracy rate of predicting CKD pathological types.

### Establishing statistical variables and databases

All results of classified or numeric variables in 588 CKD patients were input into Excel 2003 and SPSS 19.0 databases, the frequency of 40 classified variables including basic characteristics, medical history, clinical signs and laboratory data was calculated by the classification of CKD pathological types, and a special database of containing these clinical indexes was established. Following the significant difference was discriminated by the *Pearson* Chi-square (χ^2^) test or t test, the significant variables were screened and further analyzed, as shown in Supplement information.

### Data availability

All data needed to support the conclusions in this paper are present in the paper and the Supplementary information; the Supplementary Data can be downloaded from the supplements. Additional data related to this paper may be requested from the authors.

## Additional Information

**How to cite this article**: Yang, Y. *et al*. Exploration of pathological prediction of chronic kidney diseases by a novel theory of bi-directional probability. *Sci. Rep.* **6**, 32151; doi: 10.1038/srep32151 (2016).

## Supplementary Material

Supplementary Information

## Figures and Tables

**Table 1:** Frequency of clinical indexes in different CKD pathological types.

Pathological Types	Indexes	**%**	Indexes	**%**	Indexes	**%**	Indexes	**%**
MLN	Male	81.7	Duration ≤ 1Y	65.8	Oedema-F/L	59.2	Haematuria < 2Y	64.2
	Proteinuria < 2Y	80.8	High-BL	69.2	IgG-Lower	42.5	CKD-RI 4	0.8
	Haematuria ≥ 2Y	1.7	Proteinuria ≥ 2Y	1.7	High-BP	20.8	P-lower	5.0
	HB-Reduce	6.7	UBJP-Elevation	25				
FSGS	FOA.20–40Y	52.5	Duration ≤ 1Y	62.3	CKD-RI 0	36.1	Oedema-F/L	52.5
	Haematuria < 2Y	64.8	Proteinuria < 2Y	78.7	UBJP-E	60.8	CKD-RI 5/7/8	1.6
	Haematuria ≥ 2Y	8.2	Proteinuria ≥ 2Y	9.0	P-lower	6.6	K-Lower	9.8
	Fe-Lower	8.2	IgG-Lower	27.0	Tube-I/PI	24.6		
MN	FOA > 40Y	60.3	Duration ≤ 1Y	74.5	CKD-UR	38.1	Oedema-F/L	91.5
	Haematuria < 2Y	66.7	Proteinuria < 2Y	87.9	High-BL	85.1	Ca-lower	66.7
	Alb-Lower	83.0	IgG-Lower	49.6	ESR-quicken	47.5	UBJP-E	66.7
	High-DD	58.2	Tube-I/PI	54.6	FOA < 20Y	7.1	Duration > 3Y	7.8
	CKD-RI 7/8	0.7	Haematuria ≥ 2Y	0.7	Proteinuria-N	2.1	P-High	19.1
	α1Glb lower	7.1						
IgAN	Female	59.1	FOA.20–40Y	70.8	Duration ≤ 1Y	67.9	CKD-RI 1	38.0
	Haematuria < 2Y	72.3	Proteinuria < 2Y	70.1	CKD-RI 7	0	Oedema-F/L	30.7
	ESR-quicken	9.5	Haematuria ≥ 2Y	10.9	Proteinuria ≥ 2Y	11.7	High-BP	31.4
	α2Glb elevation	4.4	High-BL	41.6	Ca-lower	28.5	K-Lower	11.7
	P-lower	6.6	High-DD	14.6	HB-Reduce	8.8	UBJP-E	32.8
	Alb-Lower	31.4						
MsPGN	Female	63.0	FOA.20–40Y	55.6	Proteinuria < 2Y	66.7	IgG-Lower	11.1
	CKD-RI 4/6	0	Proteinuria ≥ 2Y	14.8	High-BP	22.2	P-lower	3.7
	α2Glb elevation	11.1	UBJP-E	25.9	Fe-Lower	3.7		
	α2Glb elevation	11.1	UBJP-E	25.9	Fe-Lower	3.7		

Notes: % indicates the occurrence frequency of indexes in CKD pathological type.

Abbreviations: Y, years; Oedema-F/L, oedema of face/lower extremity; High-BL, high blood lipid; CKD-RI 4, CKD-related inducement (kidney stones or cyst or trauma to the kidneys); High-BP, high blood pressure; P-lower, lower blood phosphorus content; HB-R, haemoglobin reduce; UBJP-E, Urine Bence-Jones Protein elevation; FOA, first onset age; CKD-RI 0, CKD-related inducement (unknown reasons); CKD-RI 5/7/8, CKD-related inducement (5: infection of urinary tract/bowel/lung or 7: thyroid disease or 8: rash/ringworm/allergic disease); K-Lower, lower blood Kalium content; Fe-Lower, lower serum Ferrum; Tube-I/PI, Urine tube/pathological tube number increase; Alb-Lower, lower albumin; ESR, erythrocyte sedimentation rate; High-DD, D-dimer high; CKD-RI 1, CKD-related inducement (cold/tonsillitis/infection of upper respiratory tract); CKD-RI 4/6, CKD-related inducement (stones/cyst/trauma of kidneys or hypertension).

**Table 2:** Verification of models of probability prediction and differential diagnosis.

Pathological types	N	Correct number	Prediction accuracy rate (%)	Pathological types of main misjudgement	Rate of false judgment (%)
MLN	34	25	73.53	MN	20.6
FSGS	23	5	21.74	MN	65.2
IgAN	30	25	83.33	MN	10.0
MN	38	38	100	—	0
MsPGN	10	0	0	IgAN/MN	50/40
Total	135	94	69.6	MN	23.1

Notes: N, number of the verified sample; MLN, mild lesion nephrosis; FSGS, focal segmental glomerular sclerosis; IgAN, IgA nephropathy; MN, membranous nephropathy; MsPGN, mesangial proliferative glomerulonephritis.
