# Supplementary material for: Exploration of pathological prediction of chronic kidney diseases by a novel theory of bi-directional probability
Source: Sci Rep. 2016 Aug 25;6:32151. doi: 10.1038/srep32151 (PMC4997332; doi:10.1038/srep32151)
Supplement: Supplementary Information [file srep32151-s1.pdf]

Exploration of pathological prediction of chronic kidney diseases by a novel theory of bi-directional probability

Yuan Yang[1,2], Min Luo[1], Li Xiao[1], Xue-jing Zhu[1], Chang Wang[1], Xiao Fu[1], Shu-guang Yuan, Fang Xiao[3], Hong Liu[1], Zheng Dong[1], Fu-you Liu[1*], Lin Sun[1*]

Supplement. Figure 1-5

Figure 1 Indexes of positive-negative bi-directional probability in MLN

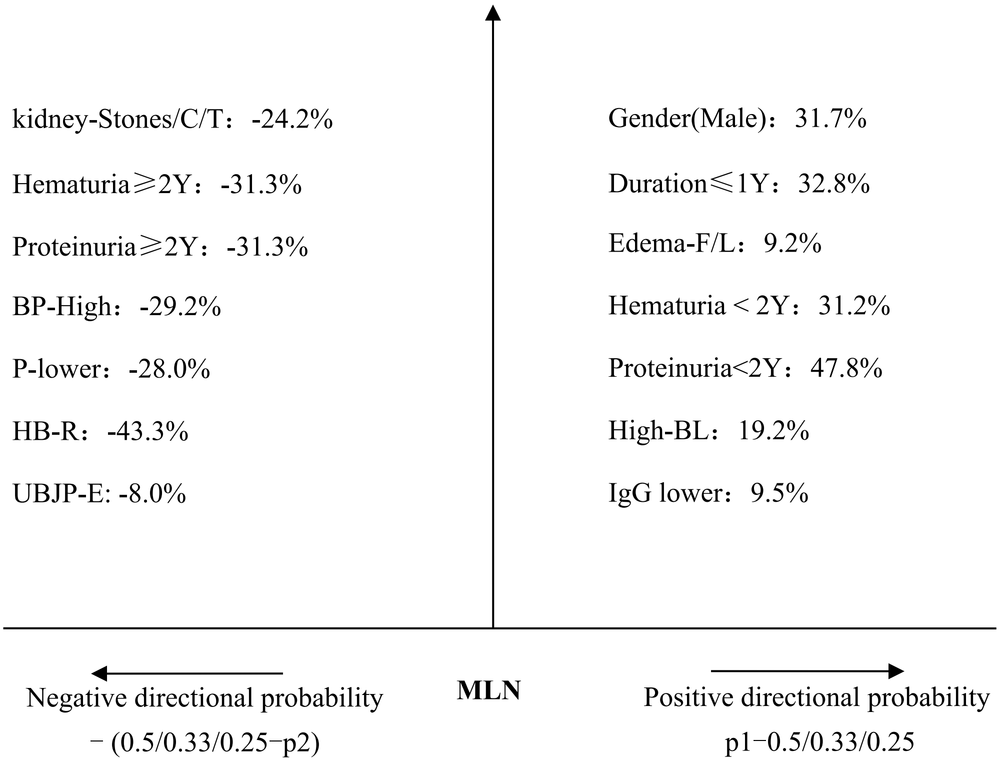

Notes: % indicates the value of positive or negative probability of indexes in MLN.

Abbreviations: Y, years; MLN, mild lesion nephrosis; kidney-Stones/C/T, CKD-related inducement (kidney stones or cyst or trauma to the kidneys); BP-High, high blood pressure; P-lower, lower blood phosphorus content; HB-R, haemoglobin reduce; UBJP-E, Urine Bence-Jones Protein elevation; Edema-F/L, oedema of face/ lower extremity; High-BL, high blood lipid.

Figure 2 Indexes of positive-negative bi-directional probability in FSGS

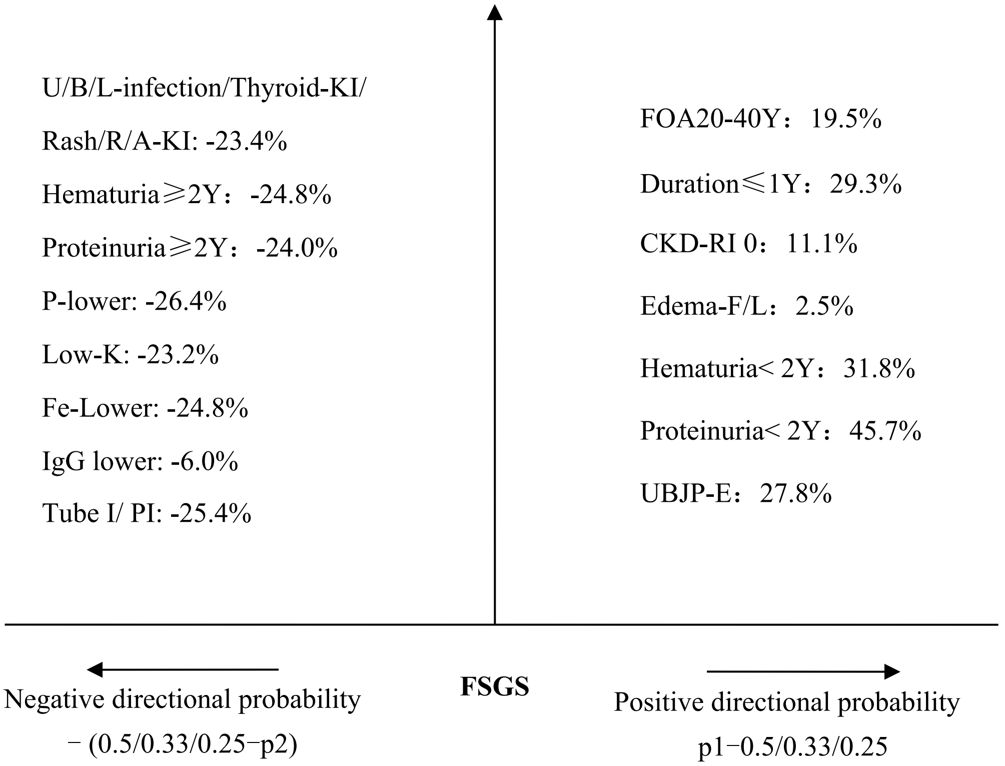

Notes: % indicates the value of positive or negative probability of indexes in FSGS

Abbreviations: FSGS, focal segmental glomerular sclerosis; U/B/L-infection/Thyroid-KI/Rash/R/A-KI, CKD-related inducement (infection of urinary tract/ bowel/ lung or thyroid disease or rash/ ringworm/ allergic disease); Low-K, lower blood Kalium content; Fe-Lower, lower serum Ferrum; Tube I/ PI, Urine tube/ pathological tube number increase; FOA20-40Y,first onset age 20-40 years; CKD-RI 0, CKD-related inducement (unknown reasons).

Figure 3 Indexes of positive-negative bi-directional probability in MN

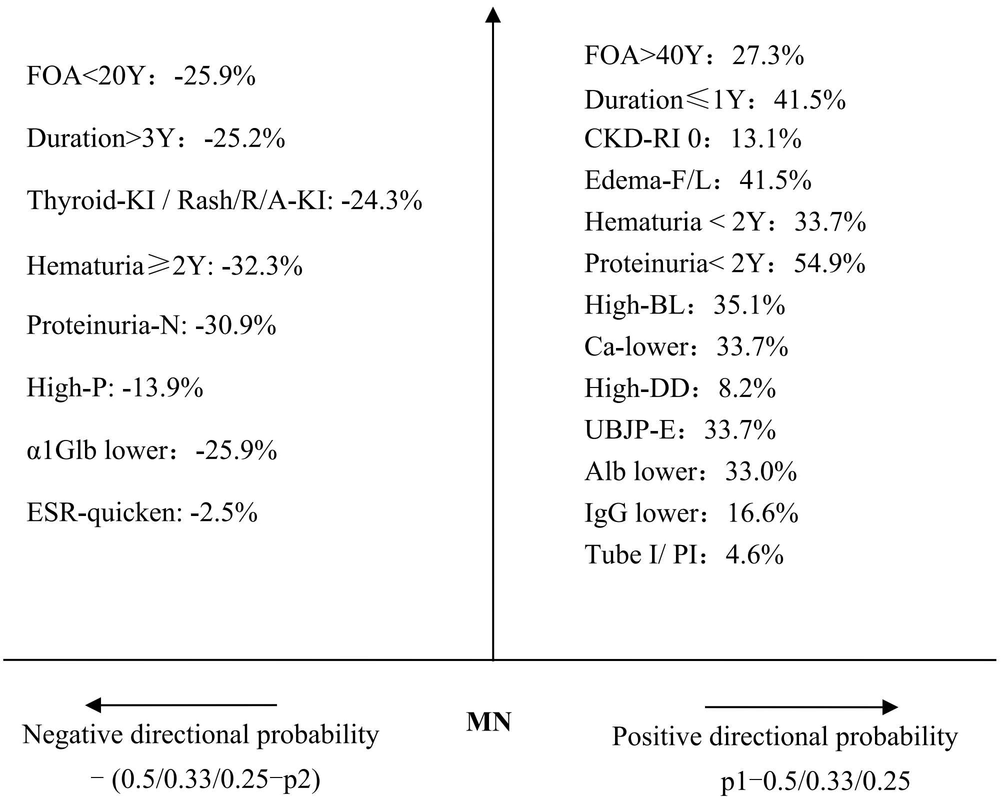

Notes: % indicates the value of positive or negative probability of indexes in MN

Abbreviations: MN, membranous nephropathy; High-P, higher blood phosphorus content; α1Glb lower, α1globulin; Ca, Calcium; ESR, erythrocyte sedimentation rate.

Figure 4 Indexes of positive-negative bi-directional probability in IgAN

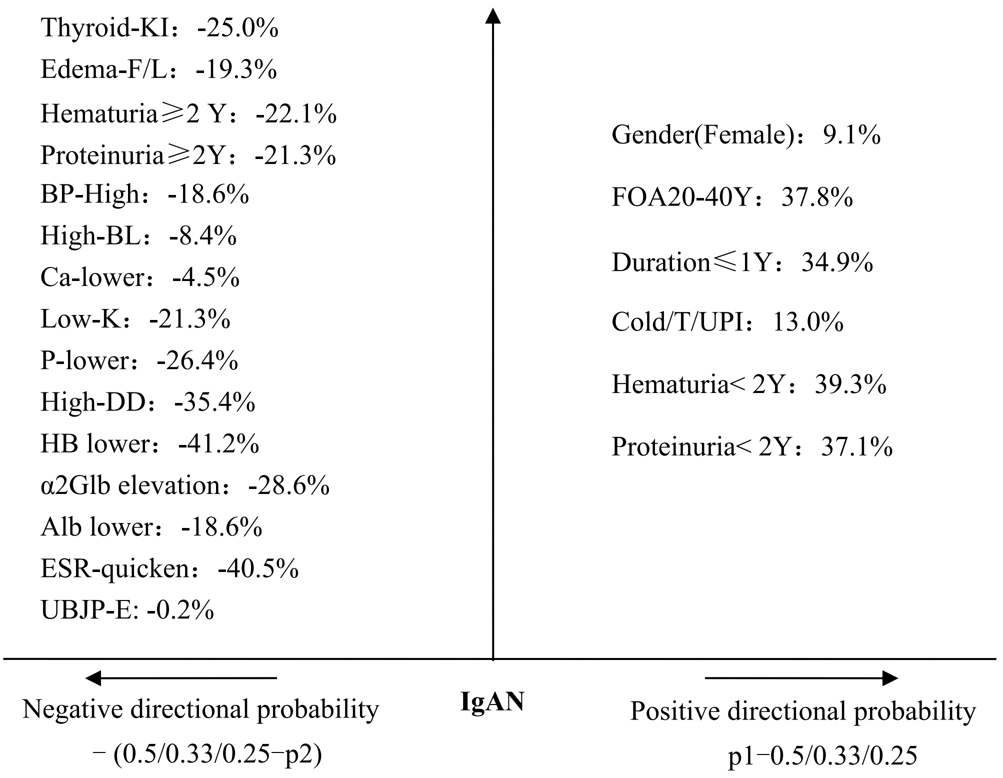

Notes: % indicates the value of positive or negative probability of indexes in IgAN

Abbreviations: IgAN, IgA nephropathy; Thyroid-KI, thyroid disease related kidney injury; High-DD, D-dimer high; Alb lower, lower albumin; Cold/T/UPI, cold/ tonsillitis/ infection of upper respiratory tract.

Figure 5 Indexes of positive-negative bi-directional probability in MsPGN

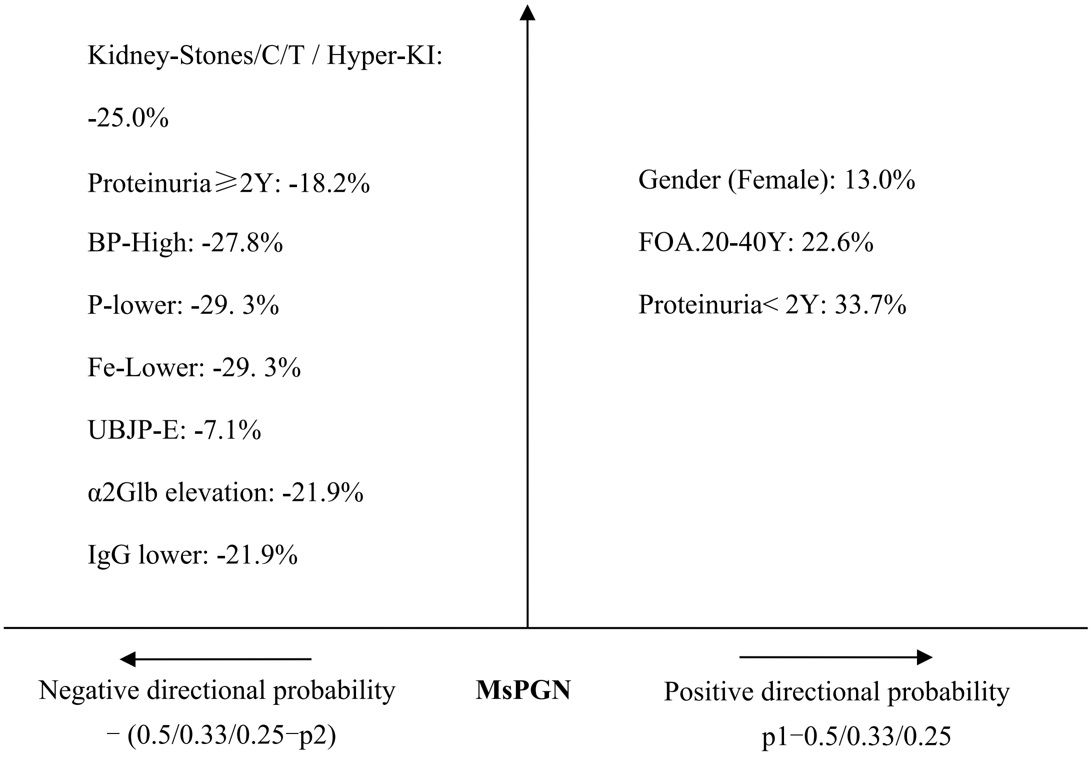

Notes: % indicates the value of positive or negative probability of indexes in MsPGN

Abbreviations: MsPGN, mesangial proliferative glomerulonephritis; Hyper-KI, hypertension related kidney injury; Others are the same as the above abbs.

Note: The following figure is not included in the model due to small sample size.

Figure. Indexes of positive-negative bi-directional probability prediction in SGN

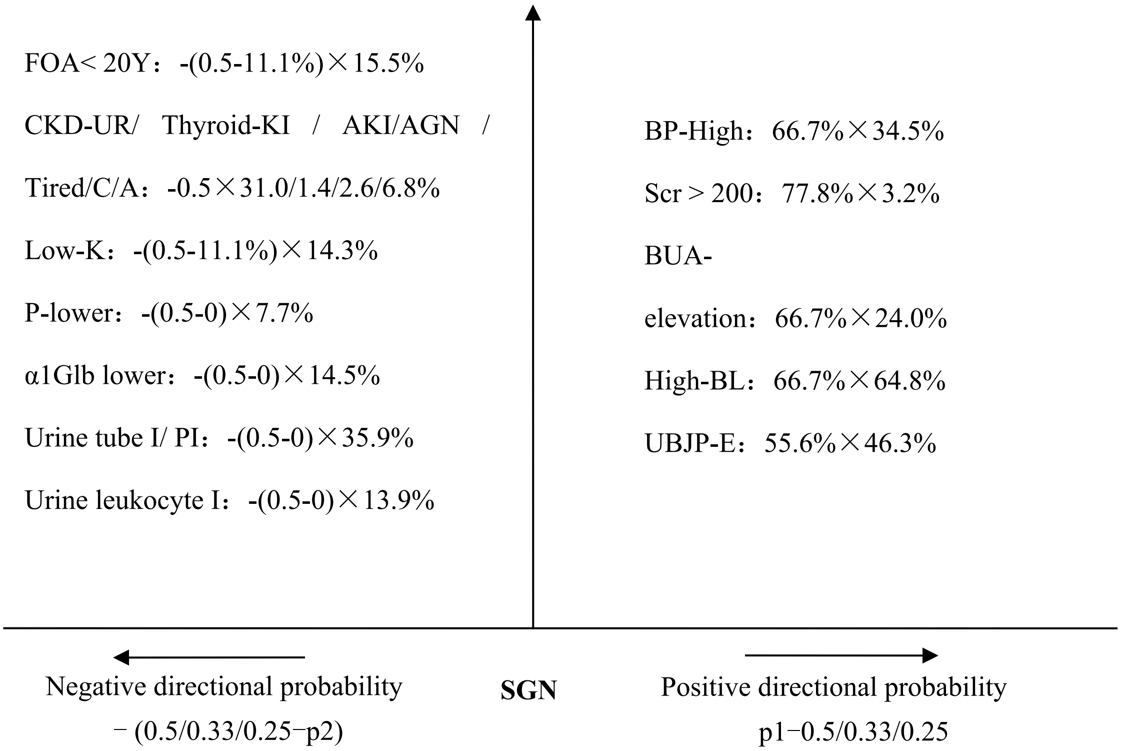

Figure 6-17 This figures were supplied in the part of results for showing the detailed OR value of Logistic regression equation of FSGS vs MLN, FSGS vs IgAN, FSGS vs MN, MLN vs IgAN, IgAN vs MN or MLN vs MN, respectively.

Figure 6 The OR value of FSGS－MLN differential diagnosis index in Logistic equation

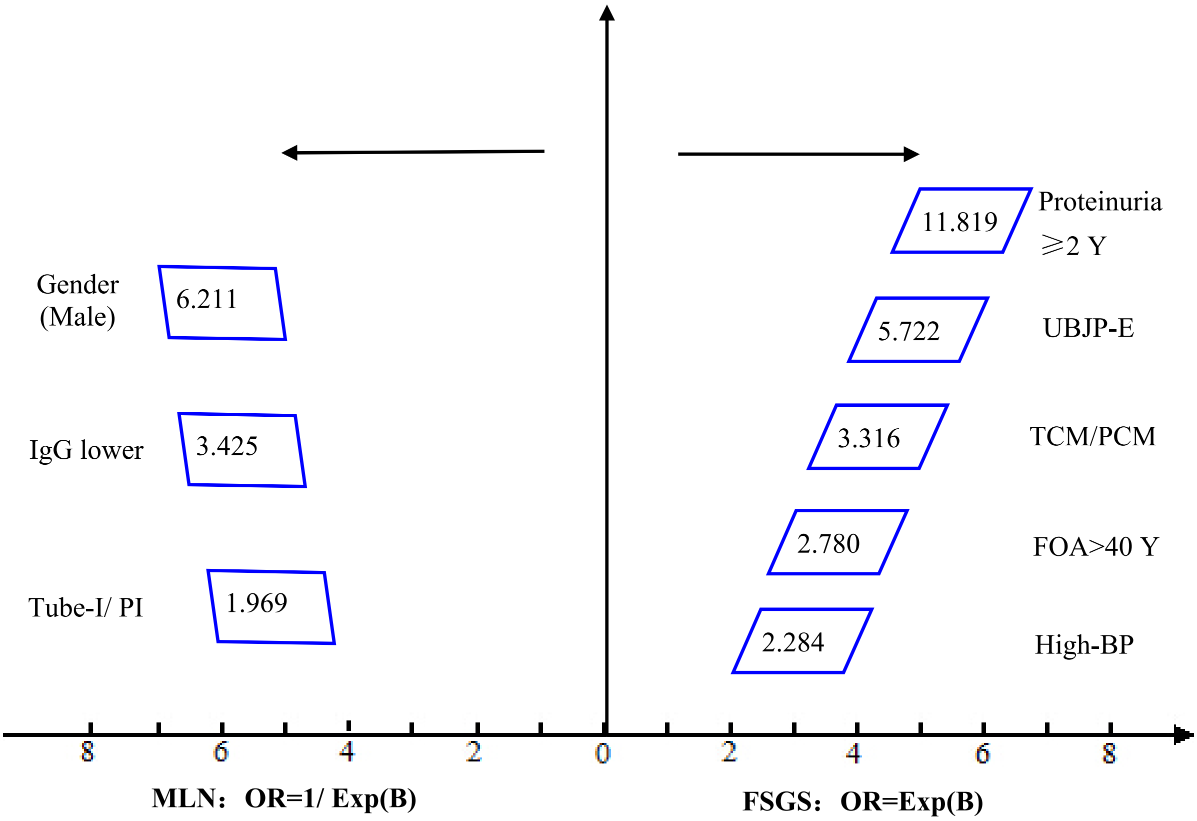

The propense factors of FSGS（Exp(B) / OR value）were that：Proteinuria≥2 Y (11.819)；UBJP-E (5.722)；TCM/PCM (3.316)；FOA > 40 Y (2.780)；BP-High (2.284)，so the result indicated the characteristic of proteinuria≥2 Y or UBJP-E or TCM/PCM or FOA > 40 Y or BP-High in CKD patients led to the increased probability of FSGS occurrence compared to that of MLN, and the increased Odds ratio（OR）values were times of 11.819, 5.722, 3.316, 2.780, 2.284, respectively.

In addition, the propense prediction factors of MLN （Exp(B) / OR value）were that：Male（0.161）；IgG lower（0.292）；Tube-I/ PI（0.508），so the result indicated the characteristic of male, IgG lower, Tube-I/ PI， in CKD patients led to the increased probability of MLN occurrence compared to FSGS, and the increased OR values were times of 6.211, 3.425, 1.969, respectively.

Figure 7 The OR value of FSGS－IgAN differential diagnosis index in Logistic equation

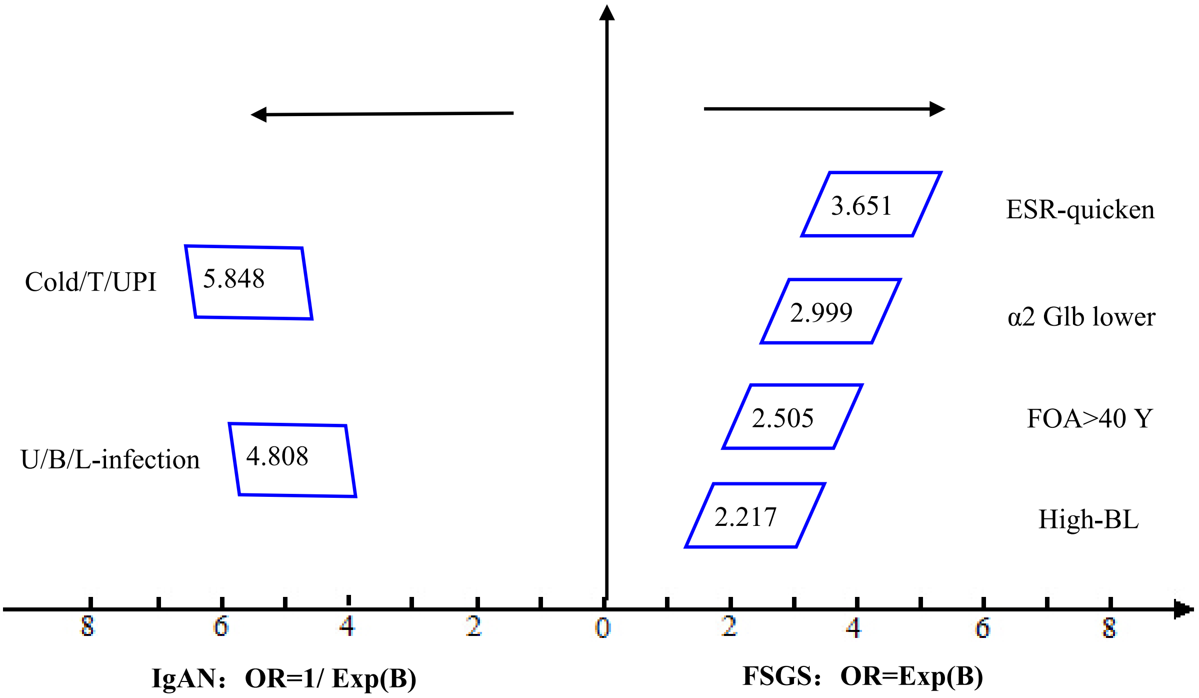

The propensity prediction factors of FSGS（OR value）were that：ESR-quicken (3.651)；α2 Glb lower (2.999)；FOA> 40Y (2.505)；High-BL (2.217)，so the result indicated the characteristic of ESR-quicken or α2 Glb lower or FOA> 40Y or High-BL in CKD patients led to the increased probability of FSGS occurrence compared to that of IgAN, and the increased OR values were times of 3.651, 2.999, 2.505, 2.217, respectively. In addition, the propensity prediction factors of IgAN（OR value）were that：The original CKD-related inducement (CKD-RI)-Cold/Tonsillitis/ Upper respiratory infection(Cold/T/UPI) (0.171）；CKD-RI-Infection of Urinary tract/Bowel/Lung (U/B/L-infection)（0.208），so the result indicated the related inducement of Cold/T/UPI and U/B/L-infection in CKD patients led to the increased probability of IgAN occurrence compared to FSGS, and the increased OR values were times of 5.848 and 4.808, respectively.

Figure 8 The OR value of IgAN－MLN differential diagnosis index in Logistic equation

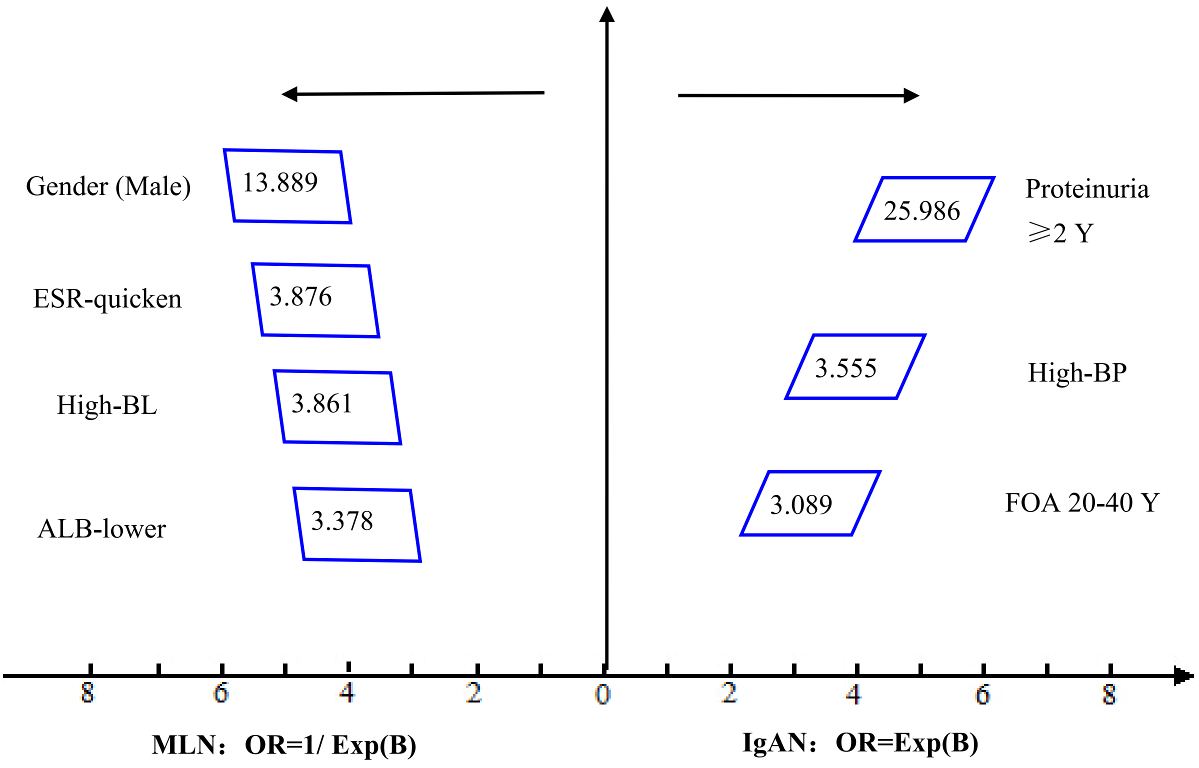

The propensity prediction factors of IgAN（OR value）were that：Proteinuria≥2 Y (25.986)；BP-High (3.555)；FOA20-40Y (3.089)，so the result indicated the characteristic of proteinuria≥2 Y or BP-High or FOA20-40Y in CKD patients led to the increased probability of IgAN occurrence compared to that of MLN, and the increased OR values were times of 25.986, 3.555, 3.089, respectively. In addition, the propensity prediction factors of MLN（OR value）were that：male (0.072)；ESR-quicken (0.258)；High-BL (0.259)；Alb-lower (0.296)，so the result indicated the characteristic of male, ESR-quicken, High-BL and Alb-lower in CKD patients led to the increased probability of MLN occurrence compared to IgAN, and the increased OR values were times of 13.889, 3.876, 3.861 and 3.378, respectively.

Figure 9 The OR value of IgAN—MN differential diagnosis index in Logistic equation

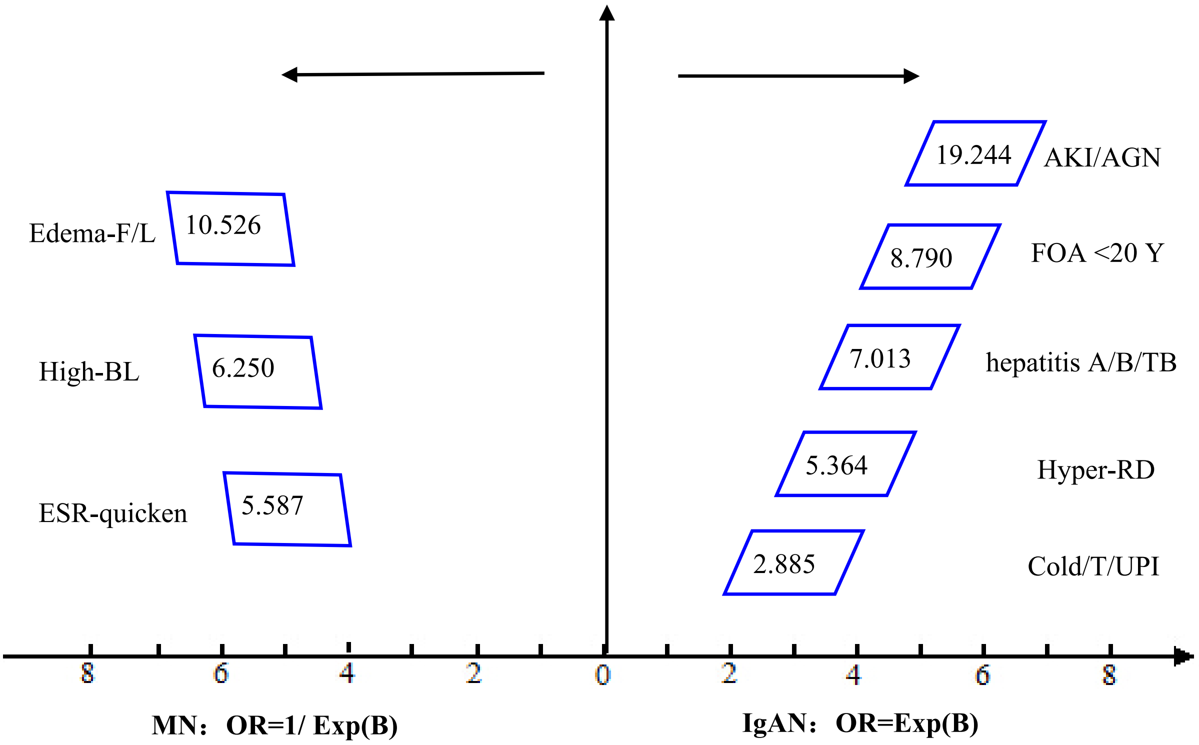

The propensity prediction factors of IgAN（OR value）were that：CKD-RI-AKI/AGN (19.244)；FOA<20Y or 20-40Y (8.790 / 7.064)；CKD-RI-hepatitis A/B/TB (7.013) or Hyper-KI (5.364) or Cold/T/UPI (2.885)，so the result indicated the related inducement of AKI/AGN, hepatitis A/B/TB, hypertension and Cold/T/UPI in CKD patients, and the characteristic of FOA<20Y or 20-40Y led to the increased probability of IgAN occurrence compared to the control of MN, and the increased OR values were times of 19.244, 7.013, 5.364, 2.885, 8.790 and 7.064, respectively. In addition, the propensity prediction factors of MN（OR value）were that：Edema-F/L (0.095)；High-BL (0.160)；ESR-quicken (0.179)，so the result indicated the characteristic of Edema-F/L, High-BL，ESR-quicken in CKD patients led to the increased probability of MN occurrence compared to IgAN, and the increased OR values were times of 10.526，6.250 and 5.587, respectively.

Figure 10 The OR value of MN—MLN differential diagnosis index in Logistic equation

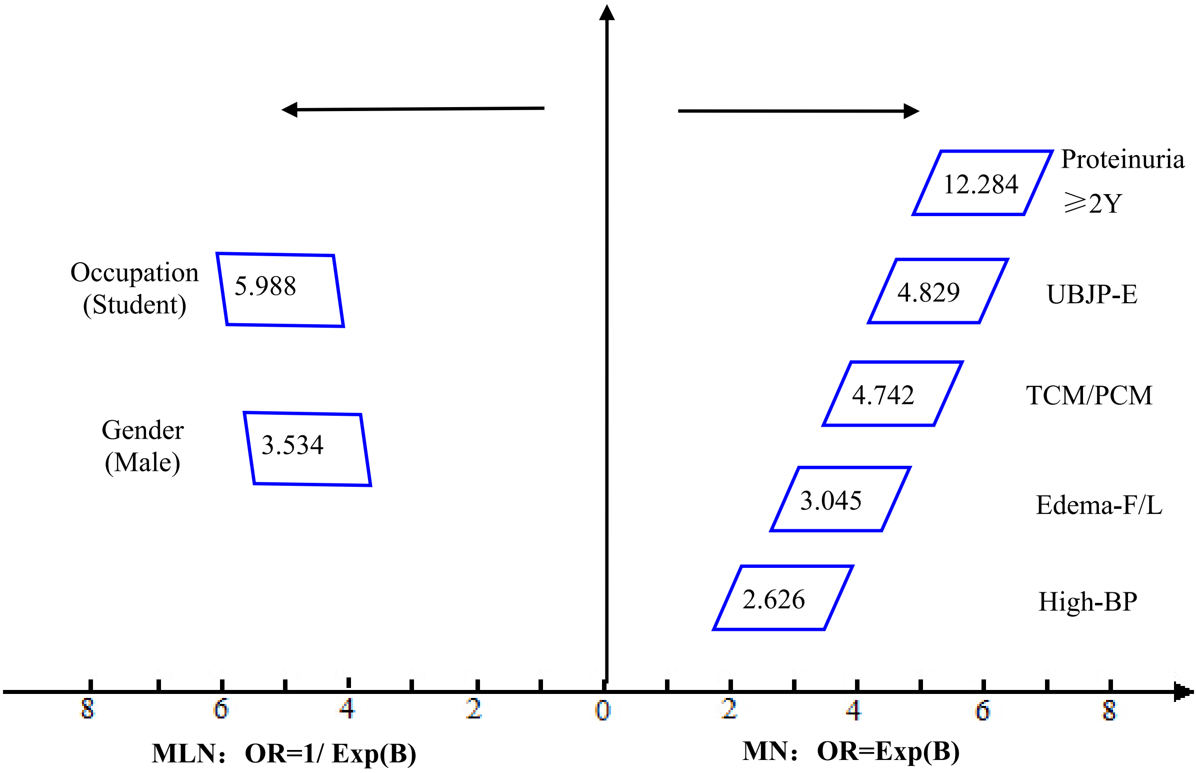

The propensity prediction factors of MN（OR value）were that：Proteinuria≥2Y (12.284)；UBJP-E (4.829)；TCM/PCM (4.742)；Edema-F/L（3.045）；BP-High（2.626），so the result indicated the characteristic of Proteinuria≥2Y, UBJP-E, TCM/PCM, Edema-F/L and BP-High in CKD patients led to the increased probability of MN occurrence compared to the control of MLN, and the increased OR values were times of 12.284, 4.829, 4.742, 3.045 and 2.626, respectively. In addition, the propensity prediction factors of MLN（OR value）were that：occupation-student（0.167）；male (0.283)，so the result indicated the characteristic of occupation-student and male in CKD patients led to the increased probability of MLN occurrence compared to MN, and the increased OR values were times of 5.988 and 3.534, respectively.

Figure 11 The OR value of MN－FSGS differential diagnosis index in Logistic equation

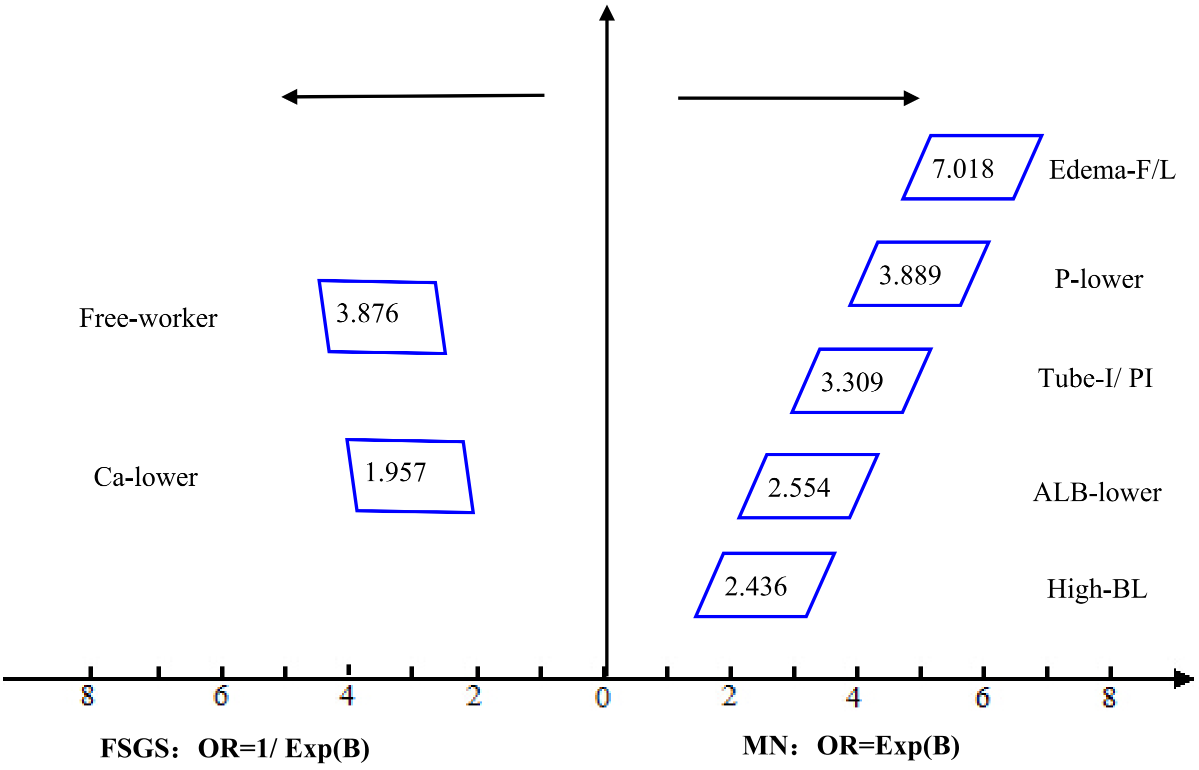

The propensity prediction factors of MN（OR value）were that：Edema-F/L（7.018）；P-lower (3.889)；Tube-I/ PI (3.309)；Alb-lower (2.554)；High-BL（2.436），so the result indicated the characteristic of Edema-F/L, P-lower, Tube-I/ PI, Alb-lower and High-BL in CKD patients led to the increased probability of MN occurrence compared to the control of FSGS, and the increased OR values were times of 7.018, 3.889, 3.309, 2.554 and 2.436, respectively. In addition, the propensity prediction factors of FSGS（OR value）were that occupation (Free-worker)（0.258） and Ca-lower (0.511)，so the result indicated the characteristic of occupation (Free-worker) and Ca-lower in CKD patients led to the increased probability of FSGS occurrence compared to MN, and the increased OR values were times of 3.876 and 1.957, respectively.

Figure 12-17 were supplied for showing the accuracy of prediction probabilities in Logistic regression models, the value of ROC curve is one-one correspondent to that of table 7.

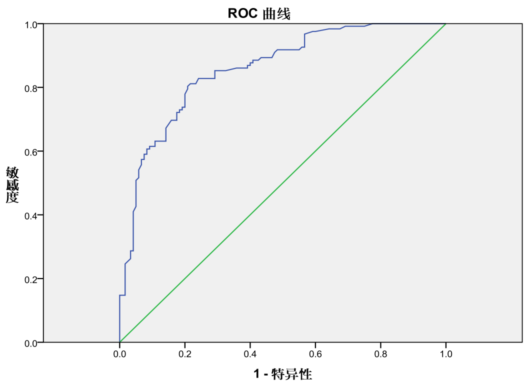

Fig.12 ROC curve evaluation of FSGS－MLN Logistic regression model

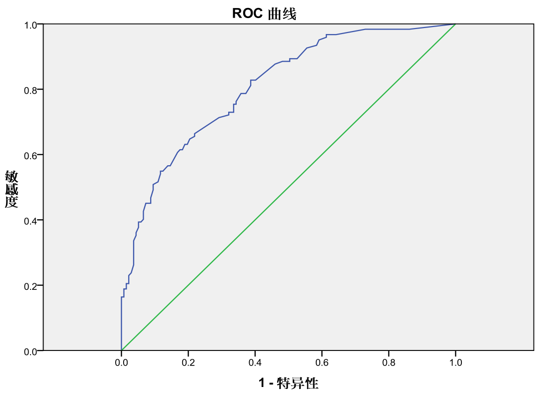

Fig.13 ROC curve evaluation of FSGS－IgAN Logistic regression model

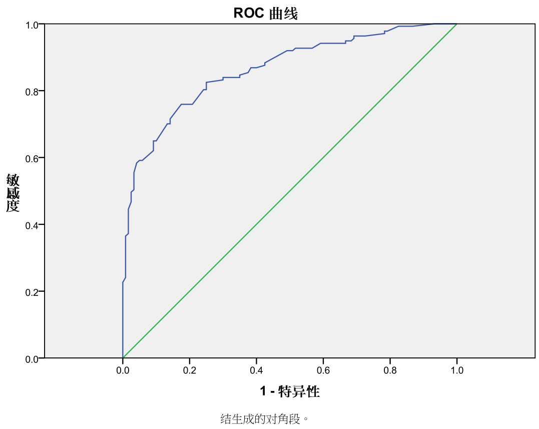

Fig.14 ROC curve evaluation of IgAN－MLN Logistic regression model

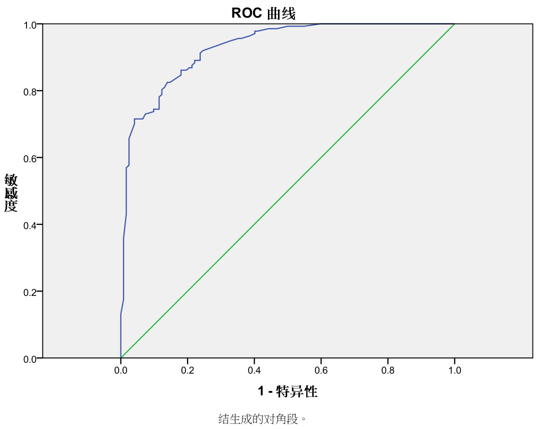

Fig.15 ROC curve evaluation of IgAN－MN Logistic regression model

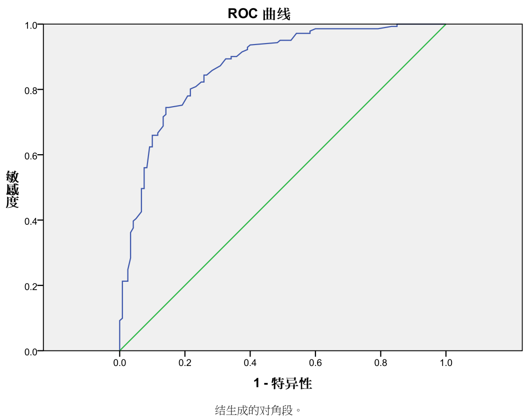

Fig.16 ROC curve evaluation of MN－MLN Logistic regression model

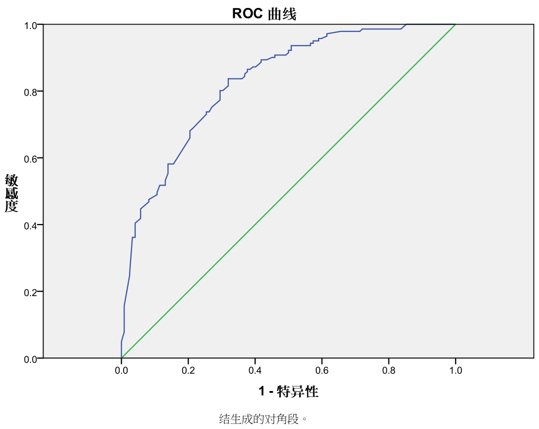

Fig.17 ROC curve evaluation of MN－FSGS Logistic regression model

Supplement. Table

Table 1-7 was supplied in the part of results for showing the detailed OR value by the Logistic regression model of differential diagnosis.

Table 1. Logistic regression model of differential diagnosis between FSGS and MLN

Variable	B	Wald χ^2	*P*	Exp(B)	95%CI
Gender (Male)	-1.825	23.612	< 0.01	0.161	0.077-0.337
FOA>40 Y	1.022	4.047	0.044	2.780	1.027-7.526
TCM/PCM	1.199	6.285	0.012	3.316	1.299-8.467
BP-High	0.826	4.629	0.031	2.284	1.076-4.846
Proteinuria≥2 Y	2.470	6.059	0.014	11.819	1.654-84.444
IgG lower	-1.232	9.425	0.002	0.292	0.133-0.641
UBJP-E	1.744	23.859	< 0.01	5.722	2.842-11.523
Tube-I/ PI	-0.678	3.398	0.065	0.508	0.247-1.044
Constant	-0.685	1.057	0.304	0.504	

Notes：B：partial regression coefficent; EXP(B)：The risk ratio value between occurrence frequency of FSGS and MLN, abbreviated to OR (below is same as previous)，EXP(B)＞1.0 indicates that the probability of FSGS occurrence increases EXP(B) times，EXP(B)＜1.0 indicates that the probability of MLN occurrence increases 1/EXP(B) times.

Table 2. Logistic regression model of differential diagnosis between FSGS and IgAN

Variable	B	Wald χ^2	*P*	EXP(B)	95%CI
FOA>40 Y	0.918	2.980	0.084	2.505	0.883-7.108
Cold/T/UPI	-1.765	16.894	0.000	0.171	0.074-0.397
U/B/L-infection	-1.568	3.480	0.062	0.208	0.04-1.083
ESR-quicken	1.295	9.838	0.002	3.651	1.625-8.200
High-BL	0.796	6.486	0.011	2.217	1.201-4.091
α2 Glb lower	1.098	5.072	0.024	2.999	1.153-7.800
Constant	-0.331	0.502	0.479	0.718	

Notes：CKD-related inducement: Cold/T/UPI and U/B/L-infection；EXP(B)＞1.0 indicates that the

probability of FSGS occurrence increases EXP(B) times，EXP(B)＜1.0 indicates that the probability of IgAN occurrence increases 1/EXP(B) times compared to the control.

Table 3. Logistic regression model of differential diagnosis between IgAN and MLN

Variable	B	Wald χ^2	*P*	EXP(B)	95%CI
Gender (Male)	-2.636	42.546	<0.01	0.072	0.032-0.158
FOA 20-40 Y	1.128	6.538	0.011	3.089	1.301-7.332
High-BP	1.268	10.020	0.002	3.555	1.621-7.795
High-BL	-1.350	13.322	<0.01	0.259	0.126-0.535
ESR-quicken	-1.355	8.170	0.004	0.258	0.102-0.653
ALB-lower	-1.216	9.503	0.002	0.296	0.137-0.642
Proteinuria ≥2 Y	3.258	10.078	0.002	25.986	3.478-194.165
Constant	1.310	5.280	0.022	3.706	

Notes：FOA: First onset age; EXP(B)＞1.0 indicates that the probability of IgAN occurrence increases EXP(B) times，EXP(B)＜1.0 indicates that the probability of MLN occurrence increases 1/EXP(B) times compared to the control.

Table 4. Differential diagnosis between IgAN and MN by Logistic regression analysis

Variable	B	Wald χ^2	*P*	EXP(B)	95%CI
FOA <20 Y	2.174	9.403	0.002	8.790	2.191-35.269
Cold/T/UPI	1.060	3.968	0.046	2.885	1.017-8.185
hepatitis A/B/TB	1.948	7.423	0.006	7.013	1.727-28.476
Hyper-KI	1.680	3.827	0.050	5.364	0.997-28.863
AKI/AGN	2.957	7.512	0.006	19.244	2.322-159.485
ESR-quicken	-1.718	11.687	0.001	0.179	0.067-0.480
Edema-F/L	-2.359	26.593	0.000	0.095	0.039-0.232
High-BL	-1.830	15.403	0.000	0.160	0.064-0.400
Constant	1.182	3.535	0.060	3.260	

Notes：CKD-related inducement: Cold/T/UPI, hepatitis A/B/TB, Hyper-KI, AKI/AGN； EXP(B)＞1.0 indicates that the probability of IgAN occurrence increases EXP(B) times compared to the control, and EXP(B)＜1.0 indicates that the probability of MN occurrence increases 1/EXP(B) times compared to the control.

Table 5. Logistic regression model of differential diagnosis between MN and MLN

Variable	B	Wald χ^2	*P*	EXP(B)	95%CI
Gender(Male)	-1.262	10.788	0.001	0.283	0.133-0.601
Occupation (Student)	-1.787	13.341	< 0.01	0.167	0.064-0.437
TCM/PCM	1.556	9.988	0.002	4.742	1.806-12.450
High-BP	0.966	6.737	0.009	2.626	1.267-5.444

Edema-F/L	1.114	6.012	0.014	3.045	1.250-7.416
Proteinuria≥2Y	2.508	4.541	0.033	12.284	1.223-123.381
UBJP-E	1.575	21.630	< 0.01	4.829	2.487-9.375
Constant	-1.637	2.991	0.084	0.195	

Notes：Y: years; TCM/PCM: Indicate that traditional Chinese medicine /proprietary Chinese medicine using before admission of Xiangya Hospital; B: regression coefficient; EXP(B) >1.0 indicates that the probability of MN occurrence increases EXP(B) times compared to the control, and EXP(B)＜1.0 indicates that the probability of MLN occurrence increases 1/EXP(B) times compared to the control.

Table 6. Logistic regression model of differential diagnosis between MN and FSGS

Variable	B	Wald χ^2	*P*	EXP(B)	95%CI
Edema-F/L	1.948	19.129	0.000	7.018	2.931-16.803
Tube-I/ PI	1.197	13.884	0.000	3.309	1.763-6.208
High-BL	0.890	5.131	0.024	2.436	1.127-5.261
P-lower	1.358	6.002	0.014	3.889	1.312-11.526
ALB-lower	0.938	5.931	0.015	2.554	1.201-5.432
Ca-lower	-0.671	3.289	0.070	0.511	0.248-1.056
Free-worker	-1.353	5.160	0.023	0.258	0.080-0.831
Constant	-2.591	20.052	0.000	0.075	

Notes：Free-worker: Occupation (Free-worker); B: regression coefficient; EXP(B) >1.0 indicates that the probability of MN occurrence increases EXP(B) times compared to the control, and EXP(B)＜1.0 indicates that the probability of FSGS occurrence increases 1/EXP(B) times compared to the control.

Table 7. Assessment of predictive ability of Logistic regression model and H-L test

Logistic regression model	Prediction accuracy rate（%）	ROC area under the curve±S.E	Asymptotic *P* values	Asymptotic 95%CI	H-L goodness of fit test *P* values
FSGS－MLN	78.5	0.858	<0.01	0.812-0.904	0.152
FSGS－IgAN	70.3	0.808	<0.01	0.757-0.860	0.470
IgAN－MLN	78.2	0.863	<0.01	0.820-0.907	0.613
IgAN－MN	83.4	0.929	<0.01	0.900-0.959	0.578
MN－MLN	78.9	0.871	<0.01	0.828-0.914	0.803
MN－FSGS	75.7	0.829	<0.01	0.780-0.878	0.905

Notes: ROC area under the curve, the area under a receiver operating characteristic curve; *P* values of H-L goodness of fit test >0.05 indicates the models are good and fit.

Table 8 The table was supplied as the part of Methods, which was used for establishing different variables from statistical viewpoint. Based on the table, a classified database can be established.

Table 8. Indexes of clinical classification in chronic kidney disease patients

NO	Indexes	Classification	Notes
**1**	Gender	0: Female； 1: Male	
**2**	Marriage	0: unmarried；1:married；2: Divorced/widowed	
**3**	Occupation	1:Farmers/unemployed; 2: worker/employee; 3: Freelance worker 4: Student 5: Health care workers	2: Include retirement
**4**	Duration (years)	0: ≤1 Y； 1: ＞1 Y, ≤3 Y； 2:＞ 3 Y	D (Y)
**5**	Frequency of admission	0: 1； 1: ≥2	
**6**	First onset age（years）	0: < 20 Y; 1: 20-40 Y; 2: >40 Y	FOA
**7**	The First time Drugs use before admission	1: Antibiotics/antiviral drugs using；2: G+/ISD；3: TCM/PCM；4: non-special drugs using	Xiangya Hospital（1,2,3）
**8**	The original CKD-related inducement	0: CKD-UR; 1：Cold/T/UPI; 2：Pregnancy/C/A; 3：hepatitis A/B/TB; 4. kidney-Stones/C/T; 5：U/B/L-infection; 6：Hypertension-KI; 7：Thyroid-KI; 8：Rash/R/A-KI; 9: AKI/AGN; 10: Tired/C/A	CKD-RI 1-10
**9**	Edema signs in CKD patients	0：none；1：Edema of Face/ Lower extremity (Edema-F/L)	
**10**	Hematuria	0：none (Hematuria-N)；1：< 2 Y; 2：≥ 2 Y (Hematuria≥2 Y)	
**11**	Proteinuria	0：none(Proteinuria-N)；1：< 2 Y; 2：≥ 2 Y (Proteinuria≥2 Y)	
**12**	Blood pressure of CKD patients	0: Normal; 1: blood pressure-High	BP-high
**13**	blood lipid (LDL/TG)	0: Normality; 1: High	High-BL
**14**	Blood sugar/glycosylated hemoglobin	0: Normality; 1: Elevation	
**15**	Alanine Aminotransferase	0: Normality；1: ALT/GPT increase	ALT-elevation
**16**	Blood urea nitrogen (2.86-7.14 mmol/L)	0: Normality; 1: Increase	BUN
**17**	Serum creatinine	0：Normality; 1: Increase	Scr

	(44-133 μmol/L)		
**18**	Blood uric acid Male:149~416uM；Female :89~357uM	0:Normality;1:Elevation（BUA-elevation）	BUA
**19**	Blood Calcium content	0: Normality；1: Lower（Ca-lower）	Ca
**20**	Blood phosphorus content	0：Normality；1：Lower (P-lower)；2：High (High-P)	P
**21**	Blood Kalium content	0: Normality；1: Lower (Low-K)；2：Increase	K
**22**	Hemoglobin	0：Normality; 1：Reduce (HB-R)	Hb
**23**	Platelet count	0：Normality; 1：Reduce; 2：High（High-PC）	PC
**24**	Blood α1-Globulin	0：Normality; 1：Decrease; 2: Increase	α1Glb
**25**	Blood α2-Globulin	0：Normality; 1：Decrease; 2: elevation（α2Glb elevation）	α2Glb
**26**	Albumin	0：Normality; 1：Decrease	Alb
**27**	Serum Ferrum	0：Normality; 1：Increase; 2: Lower	Fe-Lower
**28**	Serum Complement C3/C4	0: Normality；1: Lower; 2: Increase	C3/C4
**29**	Thrombin / activated partial thromboplastin time	0：Normality; 1：Lengthen; 2：Shorten	T/APTT
**30**	D-dimer	0: Normality; 1：High	High-DD
**31**	Erythrocyte Sedimentation Rate	0：Normality; 1：quicken(ESR-quicken)	ESR
**32**	Parathyroid hormone	0：Normality; 1：Increase; 2：Decrease	PTH
**33**	Urine bilirubin	0：Negative; 1：Positive	BIL
**34**	Urine Bence-Jones Protein (k/λ Chain)	0: Normality; 1：Increase（UBJP-E）; 2：Decrease	UBJP
**35**	Immune globulin E	0：Normality; 1：Increase	IgE
**36**	Immune globulin G	0：Normality; 1：Increase; 2：Decrease	IgG
**37**	Immune globulin A	0：Normality; 1：Increase; 2：Decrease	IgA
**38**	Urine tube /pathological tube number	0：Normality; 1：Increase	Tube-I/ PI
**39**	Urine leukocyte number	0：Normality; 1：Increase	leukocyte-I
**40**	Urine epithelium number	0：Normality; 1：Increase	epithelium-I
